# Expression of polymeric immunoglobulin receptor (PIGR) and the effect of PIGR overexpression on breast cancer cells

**DOI:** 10.1038/s41598-023-43946-6

**Published:** 2023-10-03

**Authors:** Wichitra Asanprakit, Dileep N. Lobo, Oleg Eremin, Andrew J. Bennett

**Affiliations:** 1 FRAME Alternatives Laboratory, Faculty of Medicine and Health Sciences, School of Life Sciences, University of Nottingham, Nottingham, UK; 2 Gastrointestinal Surgery, Nottingham Digestive Diseases Centre and National Institute for Health Research (NIHR) Nottingham Biomedical Research Centre, Nottingham University Hospitals NHS Trust and University of Nottingham, Queen’s Medical Centre, Nottingham, NG7 2UH UK; 3 Department of Surgery, Phramongkutklao Hospital and College of Medicine, Bangkok, Thailand; 4 MRC Versus Arthritis Centre for Musculoskeletal Ageing Research, School of Life Sciences, University of Nottingham, Queen’s Medical Centre, Nottingham, UK

**Keywords:** Breast cancer, Tumour immunology

## Abstract

Polymeric immunoglobulin receptor (PIGR) has a major role in mucosal immunity as a transporter of polymeric immunoglobulin across the epithelial cells. The aim of this study was to determine the effect of PIGR on cellular behaviours and chemo-sensitivity of MCF7 and MDA-MB468 breast cancer cell lines. Basal levels of PIGR mRNA and protein expression in MCF7 and MDA-MB468 cells were evaluated by real time quantitative polymerase chain reaction and Western blotting, respectively. MCF7/PIGR and MDA-MB468/PIGR stable cell lines, overexpressing the PIGR gene, were generated using a lentiviral vector with tetracycline dependent induction of expression. Cell viability, cell proliferation and chemo-sensitivity of PIGR transfected cells were evaluated and compared with un-transfected cells to determine the effect of PIGR overexpression on cell phenotype. The levels of PIGR mRNA and protein expression were significantly higher in MDA-MB468 cells than in MCF7 cells (380-fold, *p* < 0.0001). However, the differential expression of PIGR in these two cell lines did not lead to significant differences in chemosensitivity. Viral overexpression of PIGR was also not found to change any of the parameters measured in either cell line. PIGR per se did not affect cellular behaviours and chemosensitivity of these breast cancer cell lines.

## Introduction

Polymeric immunoglobulin receptor (PIGR) has a major role in mucosal immunity as a transporter of polymeric immunoglobulin across the epithelial cells. PIGR has been demonstrated and described as a biomarker in many cancers. The level of PIGR expression varies among cancer types and is associated with different patient outcomes and response to chemotherapy. PIGR expression in hepatocellular cancer, colon cancer, pancreatic cancer, osteosarcoma and glioma has been shown to correlate with poor prognosis^1–5^. In contrast, many studies have reported favourable outcomes with PIGR expression in patients with other cancers, including upper gastrointestinal tract, lung, endometrial, ovarian and breast cancer^6–12^.

PIGR was found to promote cellular transformation and proliferation in hepatocellular carcinoma^13^. Moreover, increasing the expression of PIGR in hepatocellular carcinoma cells can induce epithelial mesenchymal transition (EMT) of cancer cells and lead to tumour metastasis^1^. Downregulation of PIGR in pancreatic cancer cells markedly changed cellular morphology and reduced cellular proliferation, adhesion, migration and invasion^14^. In contrast, downregulation of PIGR in an endometrial adenocarcinoma cell line promoted migration of the cells although proliferation was not affected^15^. In a lung cancer cell line, overexpression of PIGR was shown to inhibit proliferation over time and in a dose-dependent manner and tended to increase apoptosis and necrosis^10^. We have previously shown that IL-1β was the M1 macrophage cytokine which enhanced PIGR expression in breast cancer cells and that IFNγ also increased PIGR expression^16^. However, the functional role of PIGR in breast cancer cells has not been elucidated.

The aim of this study was to determine the effect of PIGR on chemo-sensitivity, cell viability and cell proliferation in MCF7 and MDA-MB468 breast cancer cell lines.

## Materials and methods

### Cell lines

MCF7 (HTB-22) and MDA-MB468 (HTB-132) breast cancer cell lines were purchased from the American Type Culture Collection (ATCC, Manassas, VA, USA). A human embryonic kidney (HEK) 293FT cell line was supplied by the FRAME laboratory, University of Nottingham, UK. MCF7 cells were cultured in Eagle’s minimum essential medium (EMEM) supplemented with 10% foetal bovine serum (FBS), 0.01 mg/ml human recombinant insulin, 2 mM L-glutamine, 1 mM sodium pyruvate, 1% minimum essential medium non-essential amino acids. MDA-MB468 cells were cultured in Dulbecco’s modified Eagle medium (DMEM) supplemented with 10% FBS. HEK 293FT cells were cultured in EMEM supplemented with 10% FBS and 2 mM L-glutamine. The cells were incubated in a humidified atmosphere containing 5% CO_2_ at 37 °C.

### RNA isolation and cDNA synthesis via reverse transcription

Total RNA was isolated from the cells using TRI Reagent (Sigma-Aldrich, St Louis, MO, USA) according to the manufacturer’s instructions. Affinity-Script Multiple Temperature cDNA Synthesis Kit (Agilent Technologies, Santa Clara, CA, USA) was used to reverse transcribe 500 ng of total RNA to first-strand cDNA according to the manufacturer’s instructions.

### Real-time quantitative polymerase chain reaction (real-time qPCR)

Primers and probes sequences were designed using Primer Express Software Version 3.0 (Applied Biosystems Inc., Foster City, CA, USA https://www.thermofisher.com/uk/en/home/technical-resources/software-downloads/primer-express-software-download.html) (PIGR, forward primer: 5’ CAAGATTATCGAAGGAGAACCAAAC 3’, reverse primer: 5’ CCCGTGTTATTCCACTTGCA 3’, probe: 5’ CAAGGTCCCCTGTCACTTTCCATGCA 3’; GAPDH, forward primer: 5’ CAACAGCCTCAAGATCATCAGC 3’, reverse primer: 5’ TGGCATGGACTGTGGTCATGAG 3’, probe: 5’ CCTGGCCAAGGTCATCCATGACAA 3’). Precision FAST qPCR Master Mix (Primerdesign, Camberley, UK) and AriaMx Real-Time PCR machine (Agilent Technologies) were used to performed real-time qPCR. PIGR expressions were normalised with GAPDH gene expressions.

### Protein isolation from cell lysate and determination of concentration

Cells were washed thrice with ice cold PBS and lysed with RIPA buffer. Cell lysate was sonicated thrice on ice 10 s and incubated in a cold room on end over end rotator for 45 min. Centrifugation was performed at 21,130 *g* for 15 min at 4 °C to pellet the debris. The supernatant was collected for further analysis. Protein was quantified by Pierce BCA Protein Assay Kit (Thermo Fisher Scientific, Waltham, MA, USA) according to the manufacturer’s instructions.

### Western blotting

Protein samples were separated on a 10% SDS–polyacrylamide gel and then transferred to nitrocellulose membrane. Membrane was blocked with 5% skimmed milk in TBST for 1 h at room temperature before incubation with primary antibody (goat anti-human PIGR antibody [R&D Systems, Minneapolis, MN, USA 1:500]) overnight at 4 °C. After washing 3 times with TBST, membrane was incubated with horseradish peroxidase (HRP) conjugated secondary antibody (rabbit anti-goat IgG HRP-conjugated antibody [R&D Systems 1:1000]) for 1 h at room temperature. The membrane was washed thrice with TBST and enhanced chemiluminescent detection was performed using Immobilon Western Chemiluminescent HRP substrate (Millipore, Burlington, MA, USA) according to the manufacturer’s instructions. The chemiluminescent signal was visualized under Luminescent Image Analyzer (Fujifilm Life Science, Cambridge, MA, USA).

### Construction of inducible PIGR expression plasmids, lentiviral production and stable cell line generation

The tetracycline (Tet)-inducible pINDUCER20-hPIGR plasmids were generated using Gateway Technology with Clonase II (Thermo Fisher Scientific, Waltham, MA, USA) according to the manufacturer’s instructions. To produce lentiviral particles, HEK 293FT cells were transfected with pINDUCER20-hPIGR plasmids mixed with packaging plasmid (psPAX2, Addgene, Watertown, MA, USA) and envelope plasmid (pMD2.G, Addgene) in a 3:1 ratio of the X-tremeGENE HP DNA Transfection Reagent (Roche, Penzberg, Germany) to plasmid for 6 h. Virus containing supernatant was harvested 48 h later and filtered through a 0.45 µm filter. The lentiviral supernatant was centrifuged at 100,000 *g* (28,000 rpm) for 2 h at 4 °C to concentrate the virus. The viral pellet was resuspended in PBS and stored at 4 °C overnight. Then, 50% glycerol was added and mixed well. The virus preparation was aliquoted and stored at -80 °C. MCF7 or MDA-MB468 cells were transduced with viral concentrates for 4 h and selected by geneticin for 14 days. To induce PIGR expression, the cells were treated with doxycycline for 48 h.

Doxycycline concentrations of 20 ng/ml were selected as the low concentration and 100 and 250 ng/ml were selected as the high concentrations for MCF7/PIGR and MDA-MB468/PIGR respectively used to induce PIGR expression in the experiments. Low dose was the lowest dose that induced PIGR protein expression demonstrated by Western blotting and the high dose was the highest dose which induced PIGR protein expression to reach a plateau (the results are not demonstrated).

### Chemo-sensitivity test

MTS assay was used to evaluate the chemo-sensitivity in vitro by determining half maximal effective concentration (EC_50_) of chemotherapeutic agents for breast cancer cells. The cells were plated at a density of 5 × 10^3^ cells in each well of 96-well plates with 100 µl complete medium. Wells having only complete medium without cells were used as background controls. After overnight incubation, cell culture media was replaced with 100 µl of fresh medium with varied concentrations of chemotherapeutic agent (Doxorubicin [Selleckchem, Houston, TX, USA] or Docetaxel [Selleckchem], concentration of 0, 0.01, 0.05, 0.1, 0.5, 1, 5, 10, 25, 50 µM) and with or without doxycycline. Control wells were added with fresh medium without chemotherapeutic agent. Then the cells were incubated for 48 h. 20 µl of CellTiter 96 AQ_ueous_ One Solution Reagent (Promega, Madison, WI, USA) was added to each well. After 2 h of incubation, the absorbance at 490 nm was recorded using a spectrophotometer. Each treatment was assayed at least in quadruplicate. The results were analysed and EC_50_ values were determined using GraphPad Prism v8.1.2 for Mac (GraphPad Software, San Diego, CA, USA https://www.graphpad.com).

### Cell viability test

An MTS assay was used to determine cell viability of breast cancer cells. The method was as described in the chemo-sensitivity test but did not include the chemotherapeutic agent for cell treatment.

### Cell proliferation assay

The bromodeoxyuridine (BrdU) assay (Millipore, Burlington, MA, USA) was performed to determine proliferation of breast cancer cells. The cells were plated at a density of 5 × 10^3^ cells in each well of 96-well plates with 100 µl complete medium. Wells having complete medium without cells were used as blank control wells. After overnight incubation, 100 µl of fresh medium with or without doxycycline was gently added into each well. Fresh medium was added to control wells. After incubation for 24 h, 20 µl of diluted BrdU Reagent was added to each well and cells were incubated for a further 24 h period. The BrdU assay was performed according to the manufacturer’s instructions.

### Statistical analysis

Statistical analyses were performed with the GraphPad Prism v8.1.2 for MacOS (GraphPad Software, San Diego, CA, USA https://www.graphpad.com). The unpaired t-test was used to compare the data between two groups and one-way analysis of variance (ANOVA) was used to compare three or more groups. A probability value of less than 0.05 (2-tailed) was considered statistically significant. Multiple testing correction was performed using Bonferroni post-test correction to adjust a significant *p* value.

### Conference presentation

This paper was presented to the 2021 Annual Meeting of the Surgical Research Society and was published in abstract form: Br J Surg 2021; 108 (Suppl_5): znab282.030, https://doi.org/10.1093/bjs/znab282.030.

## Results

### MDA-MB468 cells expressed a higher level of PIGR than MCF7 cells

**Figure 1:**
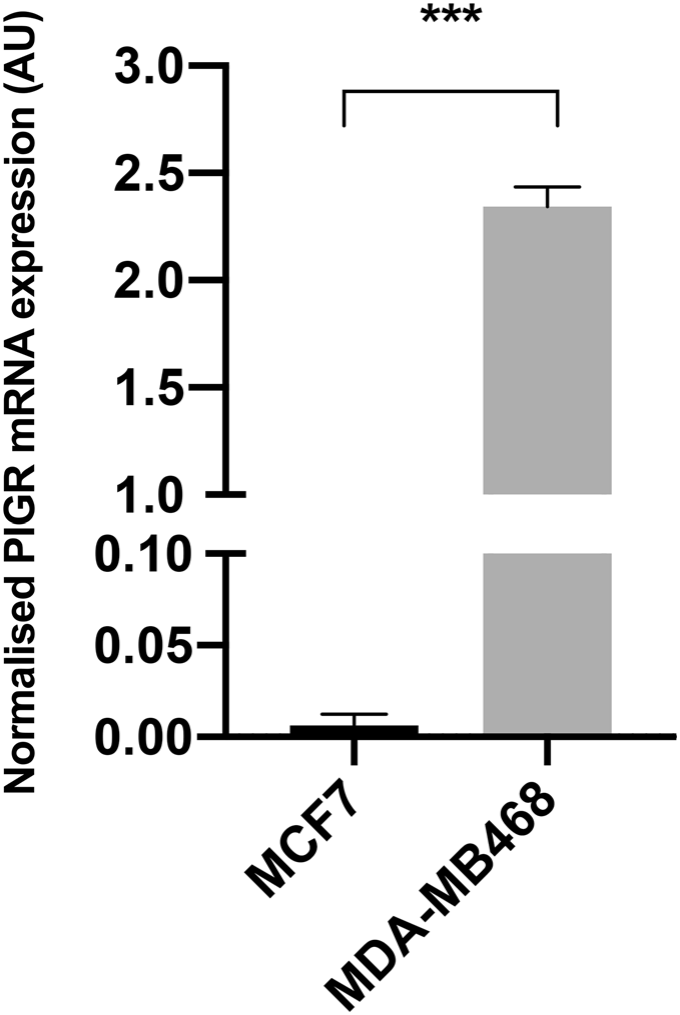
Basal expression of PIGR mRNA in MCF7 and MDA-MB468 cells. The expression of PIGR mRNA in MCF7 and MDA-MB468 cells was determined by real-time qPCR and normalised with GAPDH mRNA expression. Data are presented as mean ± SEM of three independent experiments (***p < 0.0001, unpaired t-test). AU = arbitrary units.

**Figure 2:**
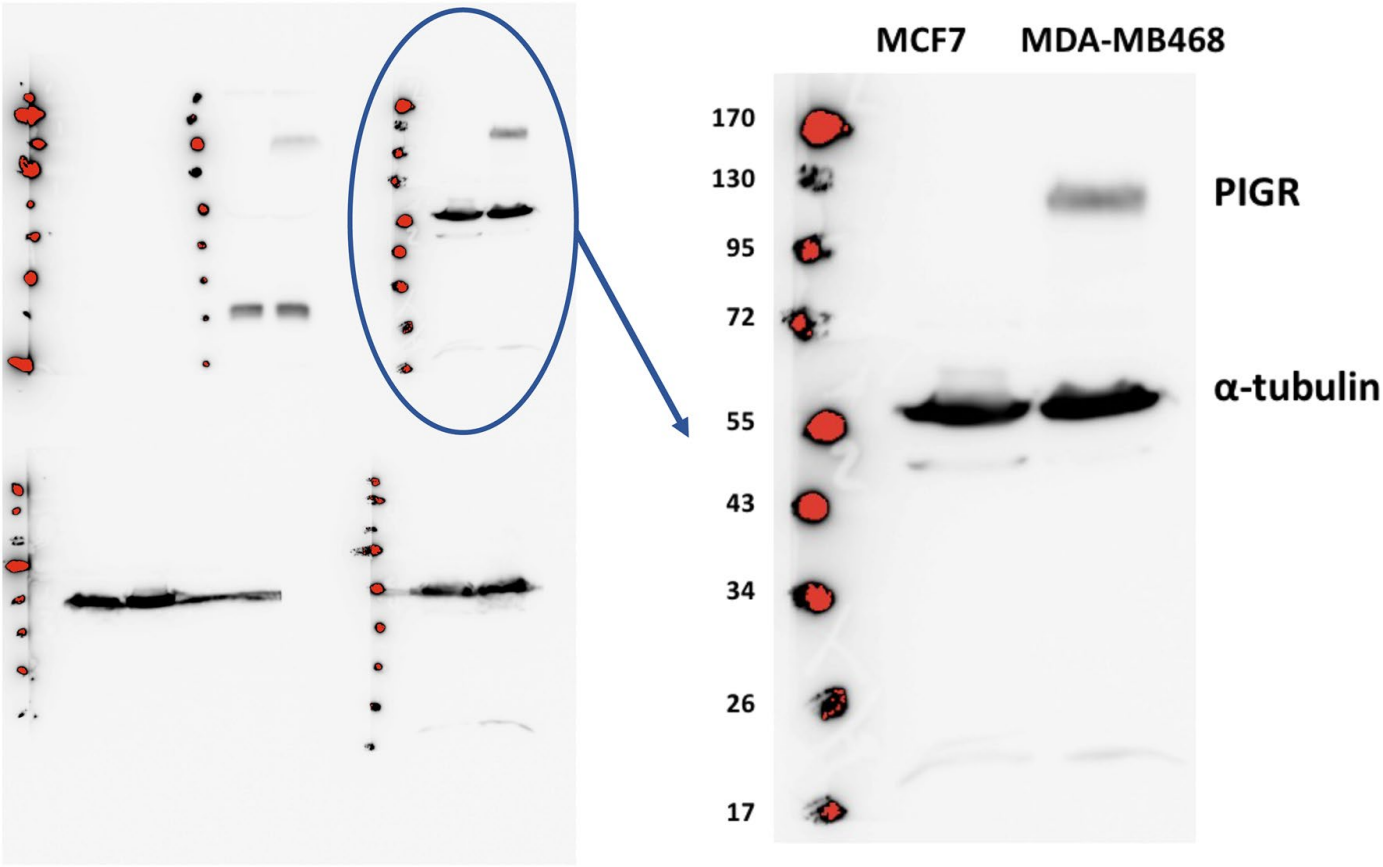
Basal expression of PIGR protein in MCF7 and MDA-MB468 cells. Representative Western blotting of three independent experiments for PIGR protein expression in MCF7 and MDA-MB468 cell lysates. 300 μg of total protein from cell lysate was loaded into each well. The blot was incubated with anti-PIGR antibody at a dilution of 1:500. α-tubulin was used as a loading control. The complete blot is on the left and the magnified blot of interest is on the right.

PIGR mRNA expression level in MDA-MB468 cells was approximately 380-fold higher than in MCF7 cells, which expressed very low levels (*p* < 0.0001) (Fig. 1). The protein expression levels which were demonstrated by Western blotting correlated with the levels of mRNA expression (Fig. 2 and Supplementary Table S1).

### MCF7/PIGR and MDA-MB468/PIGR cells were stable and overexpressed PIGR

**Figure 3:**
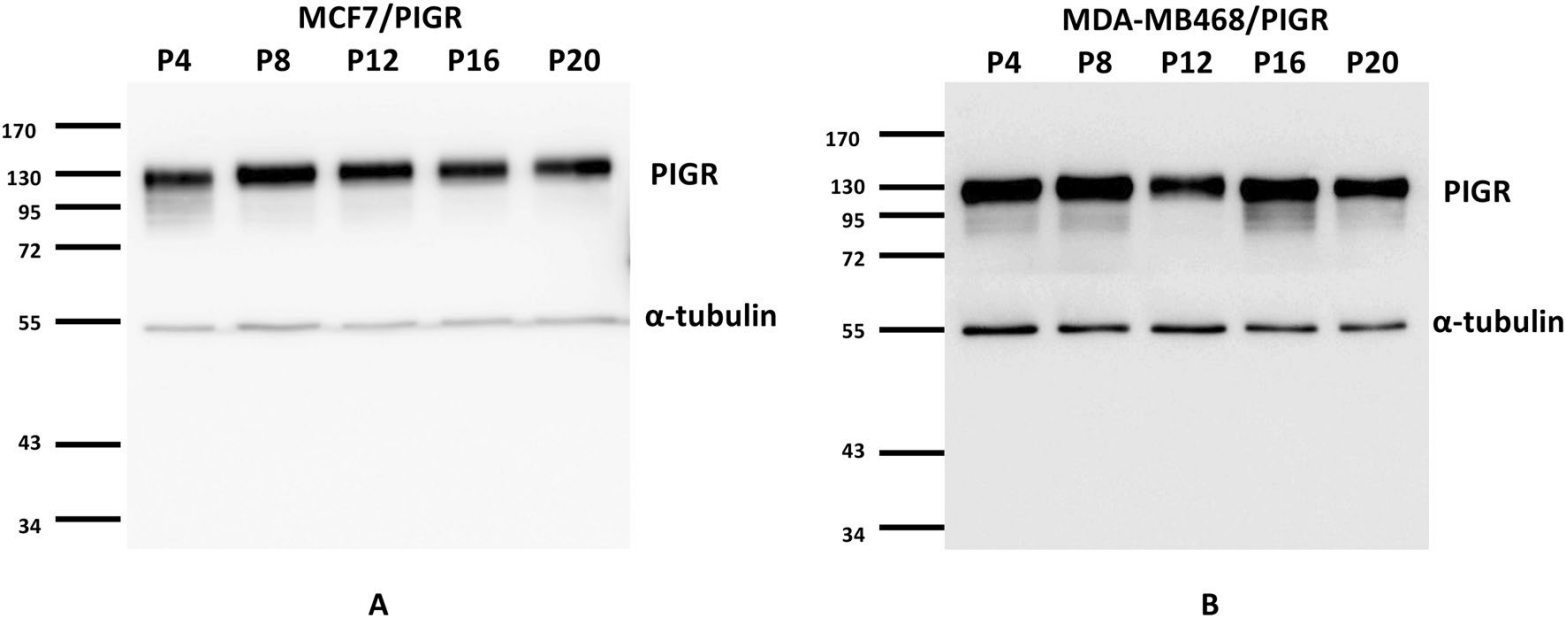
Stable PIGR overexpression in MCF7/PIGR (**A**) and MDA-MB468/PIGR (**B**) cell lines.

Generated MCF7/PIGR and MDA-MB468/PIGR stable cell lines were demonstrated to stably overexpress PIGR protein through 20 cell passages (Fig. 3).

### PIGR overexpression did not significantly change chemo-sensitivity of MCF7/PIGR and MDA-MB468/PIGR cells

Compared with MCF7 cells, MCF7/PIGR cells showed a lower EC_50_ for doxorubicin, but a higher EC_50_ for docetaxel irrespective of whether induction was carried out with doxycycline at low or high concentration (Table 1). These results indicated that MCF7/PIGR cells tended to be more sensitive to doxorubicin but more resistant to docetaxel when compared with MCF7 cells. However, the differences were not statistically significant. EC_50_ of docetaxel for MDA-MB468/PIGR cells was lower than for MDA-MB468 cells. In contrast, MDA-MB468/PIGR cells showed a higher EC_50_ for doxorubicin than MDA-MB468, whether induction was with doxycycline 20 or 250 ng/ml (Table 2). These results implied that MDA-MB468/PIGR cells tend to be more sensitive to docetaxel but more resistant to doxorubicin when compared with MDA-MB468 cells. However, the differences did not reach statistical significance.

**Table 1:** EC_50_ of doxorubicin and docetaxel for MCF7 and MCF7/PIGR cells after treatment with doxycycline 20 and 100 ng/ml.

	EC_50_ MCF7 (Mean ± SEM [µM])	EC_50_ MCF7/PIGR (Mean ± SEM [µM])	*p* value
Doxycycline 20 ng/ml			
Doxorubicin	0.989 ± 0.355	0.840 ± 0.324	0.77
Docetaxel	0.061 ± 0.015	0.069 ± 0.028	0.81
Doxycycline 100 ng/ml			
Doxorubicin	0.876 ± 0.161	0.498 ± 0.128	0.14
Docetaxel	0.046 ± 0.005	0.063 ± 0.003	0.051

Data are presented as mean and SEM of the EC_50_ of doxorubicin and docetaxel for MCF7 and MCF7/PIGR cells from three independent experiments (no statistically significant difference, unpaired t-test).

**Table 2:** EC_50_ of doxorubicin and docetaxel for MDA-MB468 and MDA-MB468/PIGR cells after treatment with doxycycline 20 and 250 ng/ml.

	EC_50_ MDA-MB468 (Mean ± SEM [µM])	EC_50_ MDA-MB468/PIGR (Mean ± SEM [µM])	*p* value
Doxycycline 20 ng/ml			
Doxorubicin	0.095 ± 0.028	0.098 ± 0.024	0.93
Docetaxel	0.037 ± 0.001	0.033 ± 0.001	0.07
Doxycycline 250 ng/ml			
Doxorubicin	0.110 ± 0.028	0.123 ± 0.008	0.68
Docetaxel	0.036 ± 0.002	0.033 ± 0.001	0.29

Data are presented as mean and SEM of the EC_50_ of doxorubicin and docetaxel for MDA-MB468 and MDA-MB468/PIGR cells from three independent experiments (no statistically significant difference, unpaired t-test).

### PIGR overexpression did not significantly change cell viability and proliferation of MCF7/PIGR and MDA-MB468/PIGR cells

**Figure 4:**
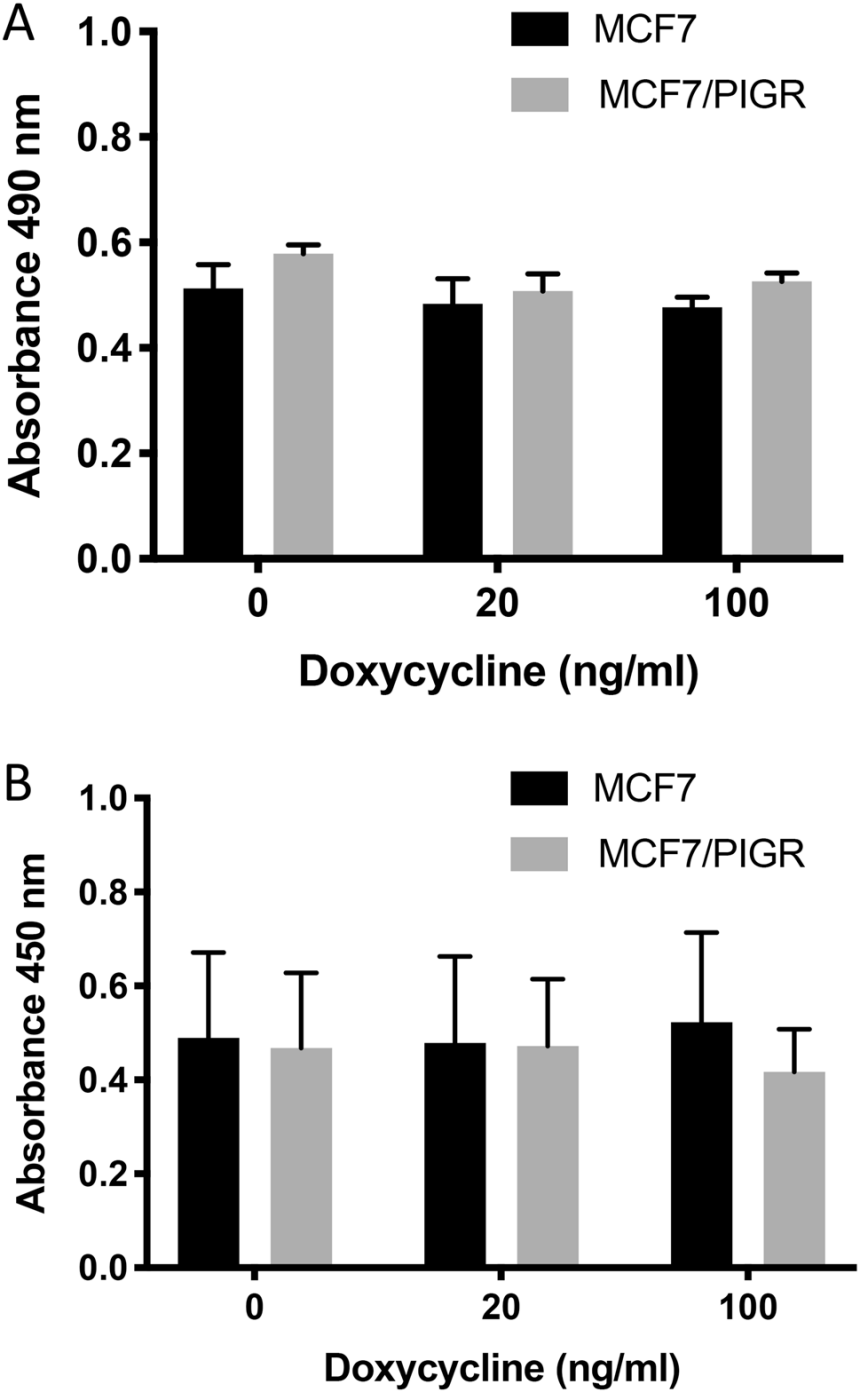
Cell viability and proliferation of MCF7 and MCF7/PIGR cells after of doxycycline induction. Panel (**A**) demonstrates absorbance at 490 nm of MTS assay which determine cell viability comparing MCF7 with MCF7/PIGR cells induced PIGR expression with doxycycline 20 or 100 ng/ml. Data are presented as mean ± SEM of three independent experiments (no statistically significant difference, one-way ANOVA with Bonferroni post-test correction). Panel (**B**) demonstrates absorbance at 450 nm of BrdU assay which determine cell proliferation comparing MCF7 with MCF7/PIGR cells induced PIGR expression with doxycycline 20 or 100 ng/ml. Data are presented as mean ± SEM of three independent experiments (no statistically significant difference, one-way ANOVA with Bonferroni post-test correction).

**Figure 5:**
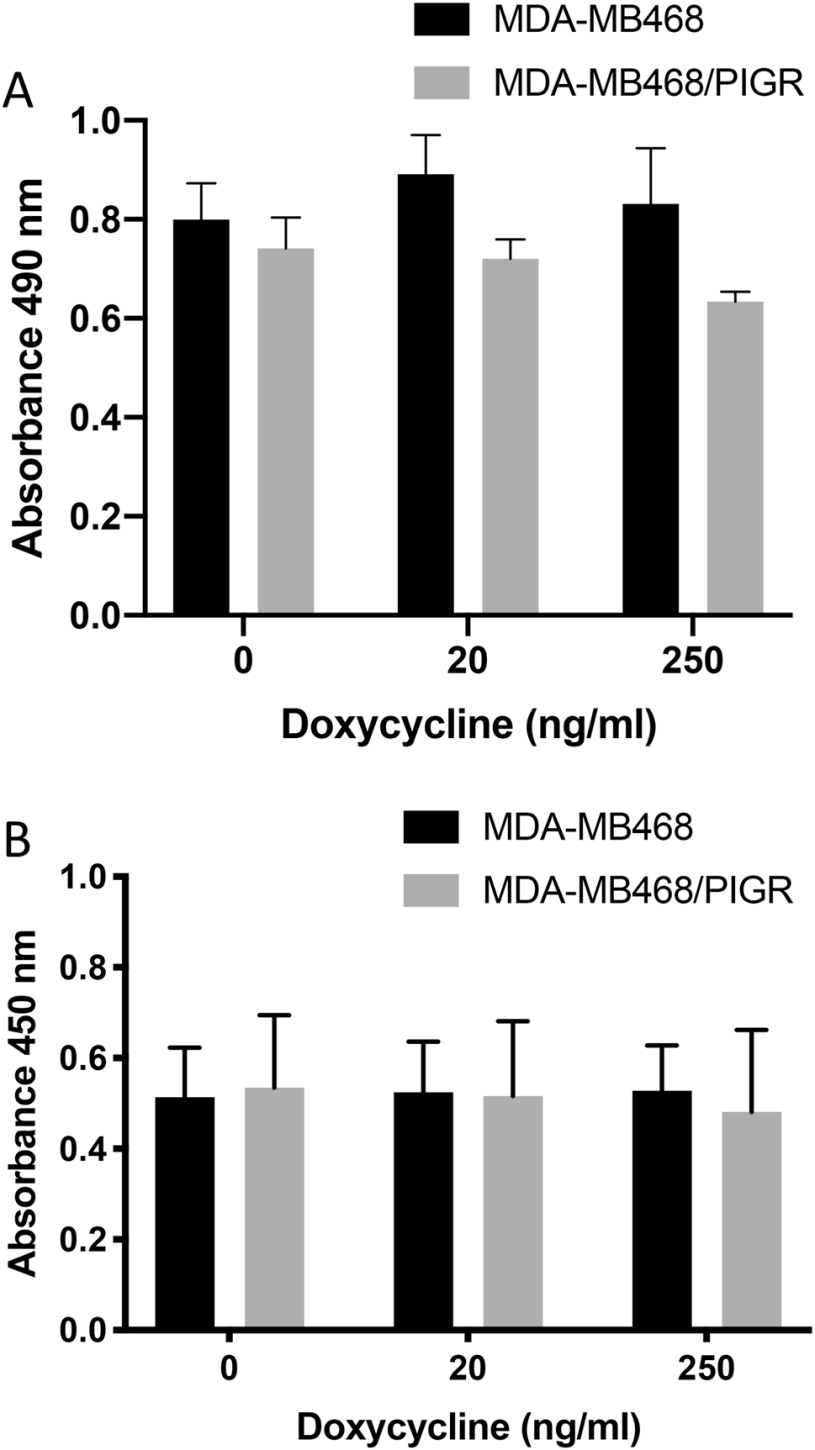
Cell viability and proliferation of MDA-MB468 and MDA-MB468/PIGR cells after doxycycline induction. Panel (**A**) demonstrates absorbance at 490 nm of MTS assay which determine cell viability comparing MDA-MB468 with MDA-MB468/PIGR cells induced PIGR expression with doxycycline 20 or 100 ng/ml. Data are presented as mean ± SEM of three independent experiments (no statistically significant difference, one-way ANOVA with Bonferroni post-test correction). Panel (**B**) demonstrates absorbance at 450 nm of BrdU assay which determine cell proliferation comparing MDA-MB468 with MDA-MB468/PIGR cells induced PIGR expression with doxycycline 20 or 100 ng/ml. Data are presented as mean ± SEM of three independent experiments (no statistically significant difference, one-way ANOVA with Bonferroni post-test correction).

PIGR overexpression did not have a significant effect on cell viability and cell proliferation in MCF7/PIGR and MDA-MB468/PIGR cells whether induction was carried out with doxycycline at low or high concentration (Figs. 4A,B and 5A,B).

## Discussion

The present study aimed to investigate the effect of PIGR expression on breast cancer cell lines with regard to chemosensitivity, cell viability and cell proliferation. MCF7 and MDA-MB468 cell lines used in in vitro studies represented the genotypic and phenotypic disparities of breast cancers. The MCF7 cell line is classified as a luminal A subtype and is representative of a chemo-resistant breast cancer. The MDA-MB468 cell line is classified as an aggressive basal-like subtype and is representative of a chemo-sensitive breast cancer^17^. Thus, these two cell lines were used to transduced *PIGR* gene and then compare the effect of PIGR on chemo-sensitivity, cell viability and cell proliferation.

The levels of PIGR mRNA expression were demonstrated to be significantly higher in MDA-MB468 cells when compared with MCF7 cells. Furthermore, PIGR protein expression was in agreement with the mRNA expression data showing that the expression was higher in MDA-MB468 than in MCF7 cells. However, the present study required a large amount of total protein (300 µg) and high concentration of primary antibody (1:500) to demonstrate PIGR expression in MDA-MB468 cells by Western blotting, while PIGR expression in MCF7 cells was not detected. This indicated that both the breast cancer cell lines studied naturally possess a low level of PIGR expression. Park et al*.*^18^ previously reported a similar finding. They used Western blotting to examine PIGR protein expression in five breast cancer cell lines, MCF7, MDA-MB231, Hs578T, SK-BR-3 and ZR-75-1, and in a normal breast cell line (Hs578Bst) and found that PIGR protein was barely detected in any of the cell lines tested. The findings from the present study demonstrated different levels of PIGR expression in distinct subtypes of breast cancer cells. This is the first time, to the best of our knowledge, that the expression of PIGR mRNA and PIGR protein in MDA-MB468 cells (basal-like subtype cell line) has been described.

To evaluate the effect of PIGR expression on chemo-sensitivity and cellular behaviour, *PIGR* gene transduction was performed in MCF7 and MDA-MB468 cells. A stable MCF7/PIGR and MDA-MB468/PIGR cell line were generated. Experiments were carried out to compare un-transduced cells with PIGR transduced cells as regards chemo-sensitivity, cell viability and proliferation. In the chemo-sensitivity assay, MCF7/PIGR cells showed a tendency to be more sensitive to doxorubicin when compared with MCF7 cells; by contrast, they were more resistant to docetaxel. Therefore, the overexpression of PIGR may confer sensitivity to doxorubicin in MCF7 cells, while on the other hand it appears to confer resistance to docetaxel. There was no statistically significant difference, however, between the EC_50_ of these two cell types. MDA-MB468/PIGR cells tended to be more sensitive to docetaxel but more resistant to doxorubicin when compared with MDA-MB468 cells. Thus, the overexpression of PIGR in MDA-MB468 may confer sensitivity to docetaxel but it appears to confer resistance to doxorubicin. This implication contrasts with the finding in MCF7 cells and may indicate a cell type specific role of PIGR. However, there was no statistically significant difference between the EC_50_ of MDA-MB468 and MDA-MB468/PIGR.

Assessment of cell viability and cell proliferation showed that there was no statistically significant difference between un-transduced and PIGR transduced cells whether MCF7 or MDA-MB468 cell lines. These results imply that increasing the expression of PIGR to the levels seen in these experiments did not affect cell viability and cell proliferation and could not be translated to significant chemo-sensitivity of MCF7 and MDA-MB468 cells.

The role of PIGR in cell proliferation in these breast cancer cell lines is in agreement with its role in endometrial cancer cells, in which alteration of PIGR does not affect cell proliferation^15^. Nevertheless, the role of PIGR in cell proliferation varies in other cancer cell types. Increasing PIGR expression can promote cell proliferation in hepatocellular carcinoma cells but can inhibit cell proliferation in lung cancer^10,13^. Reducing PIGR expression markedly decreases cell proliferation in pancreatic cancer cells^14^.

The limitation of the present study is the usage of two-dimensional (2D) monolayer cultures of breast cancer cell lines as an in vitro model of experiment. 2D cell cultures do not contain relevant components of the tumour microenvironment (TME) such as surrounding extracellular matrix, immune inflammatory cells, cytokines, oxygen and nutrient. Cancer cells in 2D culture lose their interactions between both cancer cells and non-cancerous host cells within the TME that they would have in vivo. These interactions affect cancer cell behaviour including cell viability and proliferation, response to chemotherapy, RNA and protein expression and other cellular functions^19,20^. Therefore, the studies in 2D cancer cell culture may not accurately predict responses in vivo. Despite its disadvantages, 2D cell culture is still used for cell culture research because of its simplicity and familiarity to the researchers.

## Conclusion

The present studies demonstrated that MDA-MB468 cells expressed significantly higher levels of PIGR mRNA and protein expression than MCF7 cells. Viral overexpression of PIGR was not found to change chemosensitivity, cell viability and cell proliferation in either cell line. The assumption is that PIGR per se does not affect cellular behaviour and chemosensitivity of these breast cancer cell lines in 2D cell culture. It may be a marker affected by other factors rather than being a direct contributor to the favourable outcome in patients.

### Supplementary Information

Supplementary Table S1.

## Data Availability

Data will be available on reasonable request from W. Asanprakit wichitraa@hotmail.com.

## Abbreviations

ANOVA: Analysis of variance
BrdU: Bromodeoxyuridine
DMEM: Dulbecco’s modified Eagle medium
EMEM: Eagle’s minimum essential medium
EMT: Epithelial mesenchymal transition
FBS: Foetal bovine serum
HRP: Horseradish peroxidase
IFN: Interferon
IL: Interleukin
HEK: Human embryonic kidney
PIGR: Polymeric immunoglobulin receptor
qPCR: Quantitative polymerase chain reaction
