# Supplementary material for: Expression of polymeric immunoglobulin receptor (PIGR) and the effect of PIGR overexpression on breast cancer cells
Source: Sci Rep. 2023 Oct 3;13:16606. doi: 10.1038/s41598-023-43946-6 (PMC10547702; doi:10.1038/s41598-023-43946-6)
Supplement: Supplementary file 1 — Supplementary Table S1. [file 41598_2023_43946_MOESM1_ESM.pdf]

Supplementary Data

Supplementary Table S1: PIGR and GAPDH gene expression data of MCF7 and MDA-MB468 cells from 3 independent experiments

Sample Name	Raw Ct values					
	1st Experiment		2nd Experiment		3rd Experiment	
	PIGR	GAPDH	PIGR	GAPDH	PIGR	GAPDH
MCF7-1	No Cq	16.55	No Cq	16.65	No Cq	16.72
MCF7-1	34.95	16.85	No Cq	16.65	No Cq	16.89
MCF7-1	No Cq	16.94	No Cq	16.98	No Cq	17.22
MCF7-2	35.21	17.03	35.05	16.43	No Cq	17.06
MCF7-2	35	17.07	35.36	16.52	No Cq	16.99
MCF7-2	No Cq	17.2	No Cq	16.64	No Cq	16.99
MCF7-3	34.83	16.84	No Cq	16.48	No Cq	16.93
MCF7-3	35.9	16.67	37.89	16.71	No Cq	16.88
MCF7-3	No Cq	17.1	No Cq	16.67	No Cq	17.05
MCF7-4	No Cq	16.96	36.34	16.64	37.67	16.8
MCF7-4	No Cq	16.94	No Cq	16.41	No Cq	16.75
MCF7-4	No Cq	16.96	No Cq	16.47	No Cq	17
MCF7-5	35.31	16.74	No Cq	16.58	No Cq	16.91
MCF7-5	36.88	16.52	No Cq	16.49	No Cq	17
MCF7-5	36.93	16.91	No Cq	16.56	No Cq	17.03
MCF7-6	34.63	16.49	No Cq	16.35	No Cq	16.84
MCF7-6	36.83	16.52	No Cq	16.43	No Cq	16.92
MCF7-6	No Cq	16.61	No Cq	16.54	No Cq	16.98
MDA-MB468-1	29.83	17.36	30.31	17.3	31.99	17.62
MDA-MB468-1	30.06	17.25	29.99	17.31	32.03	17.54
MDA-MB468-1	29.87	17.63	29.82	17.28	32.4	17.58
MDA-MB468-2	29.33	17.35	30.74	17.43	32.1	17.16
MDA-MB468-2	29.77	17.18	30.35	17.43	32.35	17.29
MDA-MB468-2	29.82	17.23	29.89	17.39	31.99	17.13
MDA-MB468-3	30.02	17.11	29.3	17.16	32.66	17.59
MDA-MB468-3	29.93	17.24	29.42	17.07	32.09	17.49
MDA-MB468-3	29.85	17.26	29.74	17.04	32.67	17.62
MDA-MB468-4	29.58	16.97	29.53	17.08	31.86	17.48
MDA-MB468-4	29.28	16.97	28.76	17	32.16	16.99
MDA-MB468-4	29.59	16.99	29.66	17.05	31.98	17.41
MDA-MB468-5	29.09	16.84	29.4	17.06	31.68	17.52
MDA-MB468-5	29.05	16.9	29.76	17.1	32	16.99
MDA-MB468-5	28.97	16.99	30.16	17.16	31.96	17.43
MDA-MB468-6	29.81	17.16	29.75	17.03	31.97	16.86
MDA-MB468-6	29.36	17.18	29.67	17.08	32.44	16
MDA-MB468-6	29.66	17.12	29.81	17.18	32.1	17.23

1st Experiment	PIGR	GAPDH	Normailsed PIGR
MCF7	0.000	0.162	0.000
	0.005	0.132	0.041
	0.005	0.153	0.031
	0.000	0.144	0.000
	0.003	0.168	0.015
	0.005	0.187	0.024
MDA-MB468	0.226	0.108	2.085
	0.280	0.119	2.340
	0.224	0.123	1.814
	0.311	0.142	2.185
	0.427	0.148	2.875
	0.285	0.127	2.238
**2nd Experiment**	**PIGR**	**GAPDH**	**Normailsed PIGR**
MCF7	0.000	0.152	0.000
	0.005	0.176	0.030
	0.000	0.166	0.000
	0.000	0.178	0.000
	0.000	0.174	0.000
	0.000	0.186	0.000
MDA-MB468	0.211	0.107	1.970
	0.175	0.099	1.769
	0.313	0.122	2.558
	0.366	0.126	2.901
	0.259	0.121	2.140
	0.258	0.122	2.120
**3rd Experiment**	**PIGR**	**GAPDH**	**Normailsed PIGR**
MCF7	0.000	0.151	0.000
	0.000	0.143	0.000
	0.000	0.149	0.000
	0.000	0.160	0.000
	0.000	0.147	0.000
	0.000	0.153	0.000
MDA-MB468	0.302	0.099	3.046
	0.300	0.128	2.350
	0.243	0.100	2.429
	0.331	0.121	2.742
	0.360	0.119	3.015
	0.296	0.187	1.586
